# Engaging Undergraduate Medical Students With Introductory Research Training via an Educational Escape Room: Mixed Methods Evaluation

**DOI:** 10.2196/71339

**Published:** 2025-12-08

**Authors:** Bastien Le Guellec, Victoria Gauthier, Rémi Lenain, Alexandra Nuytten, Luc Dauchet, Brigitte Bonneau, Erwin Gerard, Claire Castandet, Patrick Truffert, Marc Hazzan, Philippe Amouyel, Raphaël Bentegeac, Aghiles Hamroun

**Affiliations:** 1 Department of Public Health, Epidemiology, Health Economics and Prevention, Centre Hospitalier Universitaire de Lille, 6 rue du professeur Laguesse, Lille, 59000, France, 33 20445519; 2 Department of Neuroradiology, Centre Hospitalier Universitaire de Lille, Lille, France; 3 UMR1167 RID-AGE, Institut Pasteur de Lille, Inserm, Université de Lille, CHU Lille, Lille, France; 4 Department of Nephrology, Dialysis, Kidney Transplantation, and Apheresis, Centre Hospitalier Universitaire de Lille, Lille, France; 5 Department of Neonatology, Groupe Hospitalier de l'Institut Catholique de Lille, Lille, France; 6 Evaluation des technologies de santé et des pratiques médicales, Lille, France; 7 Department of Support for Pedagogy and Innovation (DAPI), Université de Lille, Lille, France; 8 Department of Neonatal Medicine, Centre Hospitalier Universitaire de Lille, Lille, France; 9 Faculty of Medicine, Université de Lille, Lille, France

**Keywords:** escape room, undergraduate, medical students, research, engagement, gamification

## Abstract

**Background:**

Early exposure to research methodology is essential in medical education, yet many students show limited motivation to engage with nonclinical content. Gamified strategies such as educational escape rooms may help improve engagement, but few studies have explored their feasibility at scale or evaluated their impact beyond student satisfaction.

**Objective:**

This study aimed to assess the feasibility, engagement, and perceived educational value of a large-scale escape room specifically designed to introduce third-year medical students to the principles of diagnostic test evaluation.

**Methods:**

We developed a low-cost immersive escape room based on a fictional diagnostic accuracy study with 6 puzzles mapped to five predefined learning objectives: (1) identifying key components of a diagnostic study protocol, (2) selecting an appropriate gold standard test, (3) defining a relevant study population, (4) building and interpreting a contingency table, and (5) critically appraising diagnostic metrics in context. The intervention was deployed to an entire class of third-year medical students across 12 sessions between March 2023 and April 2023. Each session included 60 minutes of gameplay and a 45-minute debriefing. Students completed pre- and postintervention questionnaires assessing their knowledge of diagnostic test evaluation and perceptions of research training. Descriptive statistics and 2-tailed paired *t* tests were used to evaluate score changes; univariate linear regressions assessed associations with demographics. Free-text comments were analyzed using the hierarchical classification by Reinert.

**Results:**

Of the 530 participants, 490 (92.5%) completed the full evaluation. Many participants had had limited previous exposure to escape rooms (206/490, 42% had never participated in one), and most (253/490, 51.6%) reported low initial confidence with critical appraisal of scientific articles. Mean overall knowledge scores increased from 62 of 100 (SD 1) before to 82 of 100 (SD 2) after the activity (+32%; *P*<.001). Gains were observed across all learning objectives and were not influenced by age, sex, or previous experience. Students rated the educational escape room as highly entertaining (mean score 9.1/10, SD 1.1) and educational (mean score 8.2/10, SD 1.5). Following the intervention, 86.9% (393/452) felt more comfortable with critical appraisal of diagnostic test studies, and 79% (357/452) considered the escape room format highly appropriate for an introductory session.

**Conclusions:**

This study demonstrates the feasibility and enthusiastic reception of a large-scale, reusable escape room aimed at teaching the fundamental principles of diagnostic test evaluation to undergraduate medical students. This approach may serve as a valuable entry point to engage students with evidence-based reasoning and pave the way for deeper exploration of medical research methodology.

## Introduction

Evidence supports the need for early exposure of medical students to research and critical appraisal of scientific articles. According to the World Federation for Medical Education 2020 standards, medical curricula must include the principles of the scientific method, cover analytical and critical thinking, medical research methodology, and evidence-based medicine [1]. The ability to interpret and apply evidence-based medicine is now widely regarded as a core competency for graduating medical students, as emphasized by both the Association of American Medical Colleges and the UK Clinical Reasoning in Medical Education group [2,3]. By developing critical appraisal skills, early exposure to research in medical education favors abilities valuable for future clinical practice (analytical reasoning and communication skills).

In particular, diagnostic test studies involve key elements of Bayesian reasoning [4], such as pretest probability, likelihood ratios, and the process of updating diagnostic probabilities based on test results—all of which are central to clinical decision-making [5]. However, evidence shows that medical professionals struggle with these concepts [6]. In addition, integration of such nonclinical skills into medical curricula is arduous, especially with undergraduate students [7,8]. In particular, generating and maintaining student interest is highly challenging [9]. In France, for example, critical appraisal of scientific articles, despite its inclusion in the final undergraduate national matching exam since 2009, varies in terms of course load and content across universities, and medical students lack motivation to invest time in nonclinical skills [10]. However, as observed with the recent expansion of biomedical literature related to the COVID-19 pandemic, physicians are at the center of both scientific and societal discussions involving critical appraisal of medical literature [11]. Thus, innovative strategies are needed to engage medical students with research training and critical appraisal of scientific articles early in their curriculum [7].

Educational escape rooms (EERs) have recently garnered growing interest in health professional education [12-15]. These gamified, immersive scenarios typically involve a series of puzzles that participants solve collaboratively, each aligned with a specific learning objective. While most published studies have focused on fostering clinical reasoning or teamwork, only a few have examined the potential of EERs for teaching research methodology [16,17]. Existing evidence remains limited, often based on small-scale initiatives with variable outcomes [12,18-20]. This gap underscores the need to explore their applicability in large cohorts and in domains beyond clinical knowledge.

This study evaluated the feasibility and perceived educational value of a large-scale EER designed to introduce medical students to key aspects of scientific methodology. The pedagogical content focused specifically on the evaluation and interpretation of diagnostic test studies while also aiming to foster teamwork and engage students with a nonclinical topic early in their training.

## Methods

### Escape Room Design and Learning Objectives

Using guidelines from Davis et al [14], we developed an EER for third-year medical students with three main goals: (1) to introduce fundamental principles of research methodology and terminology, (2) to promote collaborative problem-solving through teamwork, and (3) to foster more positive attitudes toward research training. The design team included 4 medical doctors, 1 pharmacist, and 1 medical resident, drawing on interdisciplinary expertise in clinical medicine, public health, and pedagogy.

A diagnostic accuracy study was deliberately chosen as the pedagogical framework as this type of research offers an accessible and clinically meaningful entry point for undergraduate students without previous exposure to research methods. It aligns closely with diagnostic reasoning processes familiar to most learners while also serving as a structured introduction to key methodological concepts—such as reference standards, population selection, and diagnostic performance metrics (eg, sensitivity and specificity)—that remain challenging even for many practicing clinicians [5,6]. By anchoring the learning objectives in a framework that is both practical and conceptually rich, this approach facilitates early engagement with evidence-based thinking without requiring advanced statistical background.

Learning objectives were defined a priori based on recurrent misconceptions observed in upper-year students and the collective teaching experience of the faculty involved. By the end of the session and debriefing, students were expected to (1) identify the key components of a diagnostic accuracy study protocol, including relevant methodological tools; (2) recognize the characteristics of an ideal reference standard, including performance, cost, invasiveness, and availability; (3) understand the structure of a study population that includes both individuals with and without the condition of interest; (4) construct and interpret a contingency table from diagnostic test and gold standard results; and (5) critically assess the strengths and limitations of a diagnostic test based on its performance metrics and intended clinical use.

### Escape Room Scenario

**Figure 1.**
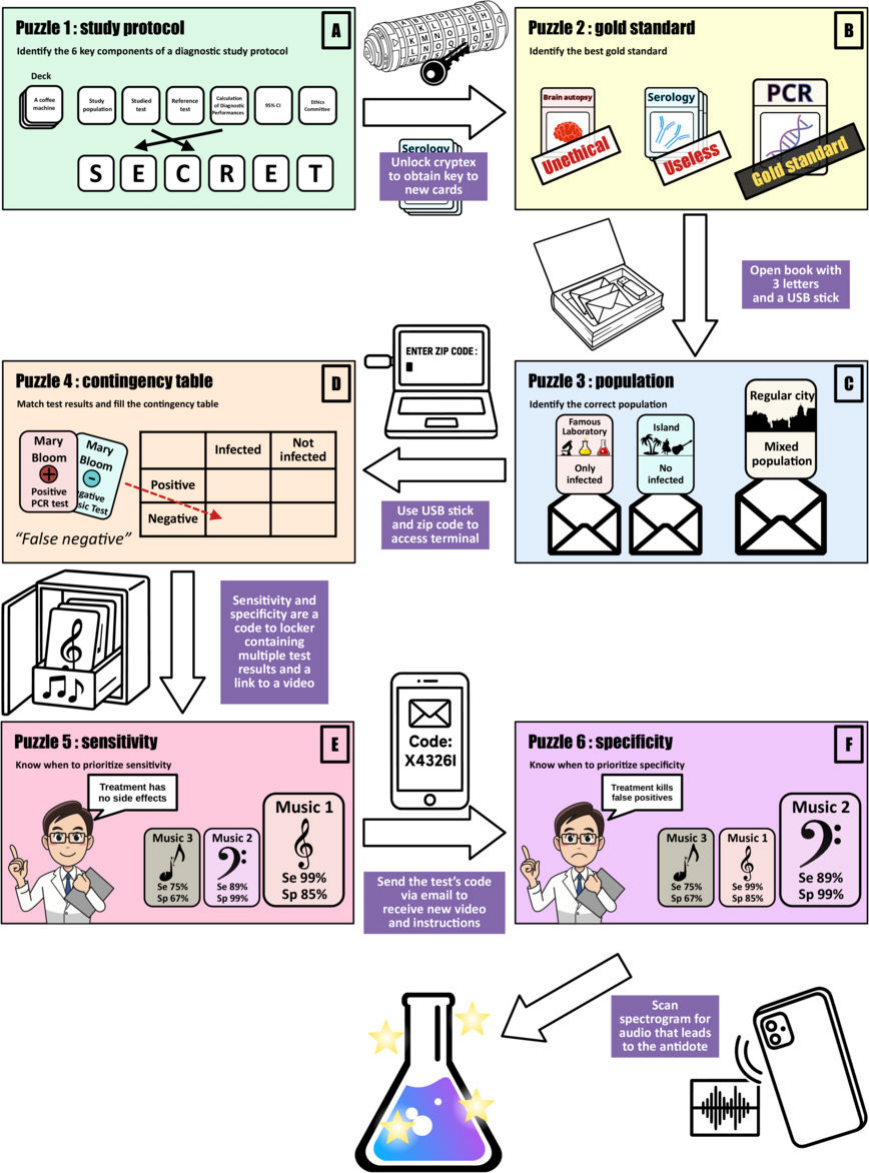
Narrative sequence and pedagogical content of the 6 puzzles composing the educational escape room. (A) Puzzle 1—study protocol. Participants select the 6 key components required to design a diagnostic accuracy study from a deck of 24 color-coded sticky notes. Each correct sticky note reveals 1 letter; when combined, they form a password that unlocks a cryptex. (B) Puzzle 2—gold standard. Inside the cryptex, participants find a key granting access to a new set of test cards. They must identify the most appropriate reference test. Although brain autopsy provides definitive results, it is excluded as unethical and impractical. Serology is dismissed due to poor diagnostic value. Polymerase chain reaction (PCR) is correctly selected as a feasible and valid gold standard. A clue hidden in invisible ink on the back of the PCR card leads them to the next step. (C) Puzzle 3—study population. Inside a hollow book designed as a lockable book safe, participants find a USB stick and 3 fictional letters each proposing a different study population: only infected individuals (famous laboratory), only uninfected individuals (isolated island), and a mixed population (regular municipality). Despite the laboratory’s prestige, they must reject biased populations and select the mixed group to ensure valid assessment of diagnostic performance. (D) Puzzle 4—test results and matching. Using the zip code from the city letter and the USB stick, participants access a computer terminal. They learn that the studied test involves a music-based rhythm activity. They receive patient test results, one card per test per patient, and must match each pair. Recognizing that both the studied test and reference test are needed for each patient, they correctly populate the contingency table to proceed. They calculate sensitivity (Se) and specificity (Sp), which together form the code to open a locker. (E) Puzzle 5—sensitivity. Inside the locker, they find test cards associated with musical rhythms, each labeled with sensitivity and specificity values. A QR code on one card links to a researcher’s video revealing that the treatment has no side effects. Participants must deduce that false positives are acceptable and, therefore, select the test with the highest sensitivity. They email the chosen test’s code to proceed. (F) Puzzle 6—specificity. A second video reveals a crucial update: the treatment is actually harmful to false positives. Participants must now prioritize specificity to avoid treating uninfected individuals. They scan a spectrogram printed on the back of each card using a mobile app. The correct choice plays a final audio message and leads to the antidote and a reward.

The escape room scenario centered on a fictional outbreak of a zombie virus disease. Students were tasked with identifying an effective diagnostic test to detect infected individuals and locate an antidote. Working in teams, they had 60 minutes to examine the research notes of a mysterious scientist who had nearly discovered a high-performing test. To succeed, students needed to solve 6 sequential puzzles, each aligned with one of the predefined learning objectives (Figure 1).

The six puzzles included (1) classifying keywords relevant to diagnostic study protocols (*study protocol*), (2) selecting the most appropriate gold standard test from a series of candidates (*gold standard*), (3) identifying a valid study population using fictional application letters (*study population*), (4) analyzing mock laboratory test results to construct a contingency table (*contingency table*), (5) calculating diagnostic accuracy metrics based on the table (*test metrics*), and (6) interpreting these metrics to choose the best test in context (*metric appraisal*). The terms in parentheses will be used throughout the manuscript for clarity.

**Figure 2.**
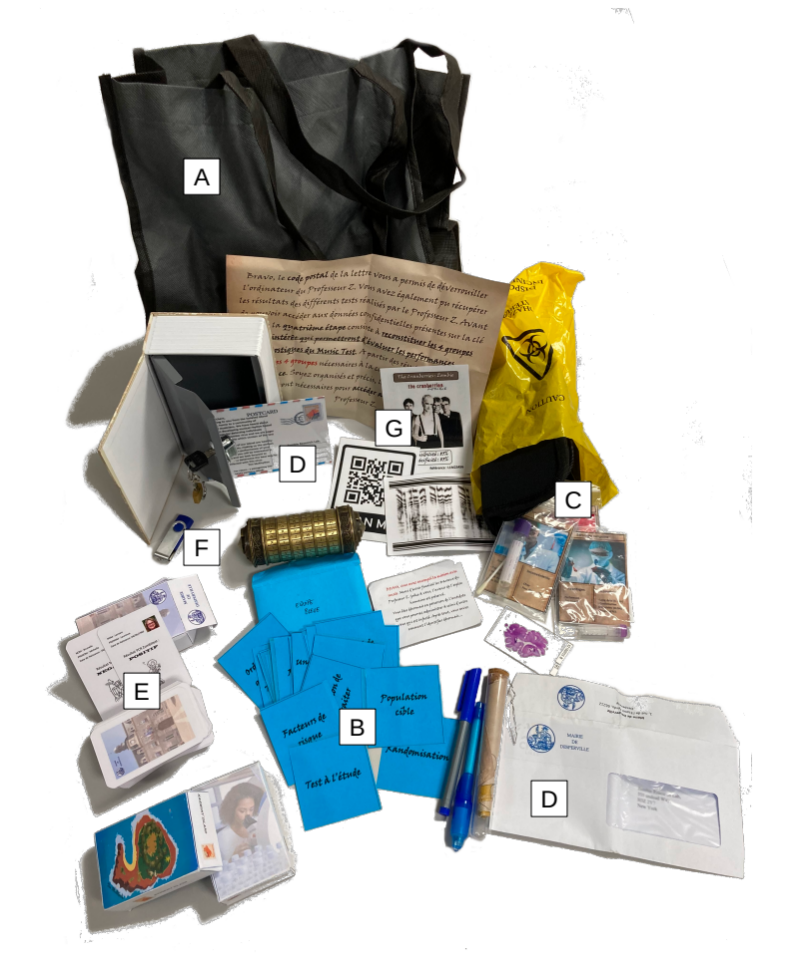
Accessories used throughout the educational escape room. (A) All materials are stored in a single portable bag to ensure easy deployment. (B) Keyword cards used to reconstruct the components of a diagnostic accuracy study (puzzle 1, *study protocol*). (C) Candidate reference tests from which participants must identify the most appropriate gold standard (puzzle 2, *gold standard*). (D) Fictional letters describing different populations, among which students must select the most suitable study population (puzzle 3, *study population*). (E) Decks of index and reference test result cards used to reconstitute patient data pairs (puzzle 4, *contingency table*). (F) USB flash drive containing an automated program through which students must input their reconstructed contingency table (puzzle 5, *test metrics*). (G) Test cards with various diagnostic performance metrics from which students must select the most appropriate test depending on the clinical scenario (puzzle 6, *metric appraisal*).

Each completed puzzle provided a clue, object, or code that allowed progression to the next stage of the game. A team succeeded when they completed all 6 puzzles within the allotted time, thereby unlocking the location of the antidote. The scenario was fully autonomous: game masters were available only to provide hints or intervene in case of technical difficulties upon request. The immersive experience included props such as a cryptex, mock test description cards, a book safe, invisible ink, a digital lockbox, a custom-designed computer program, role-play videos, QR codes, a spectrogram decryption smartphone app, and purpose-built board game–style cards (Figure 2). All materials were original creations funded by the Faculty of Medicine at the University of Lille, with a total cost of €1800 (approximately US $1925) for approximately 600 students.

### Setting, Materials, and Staffing

**Figure 3.**
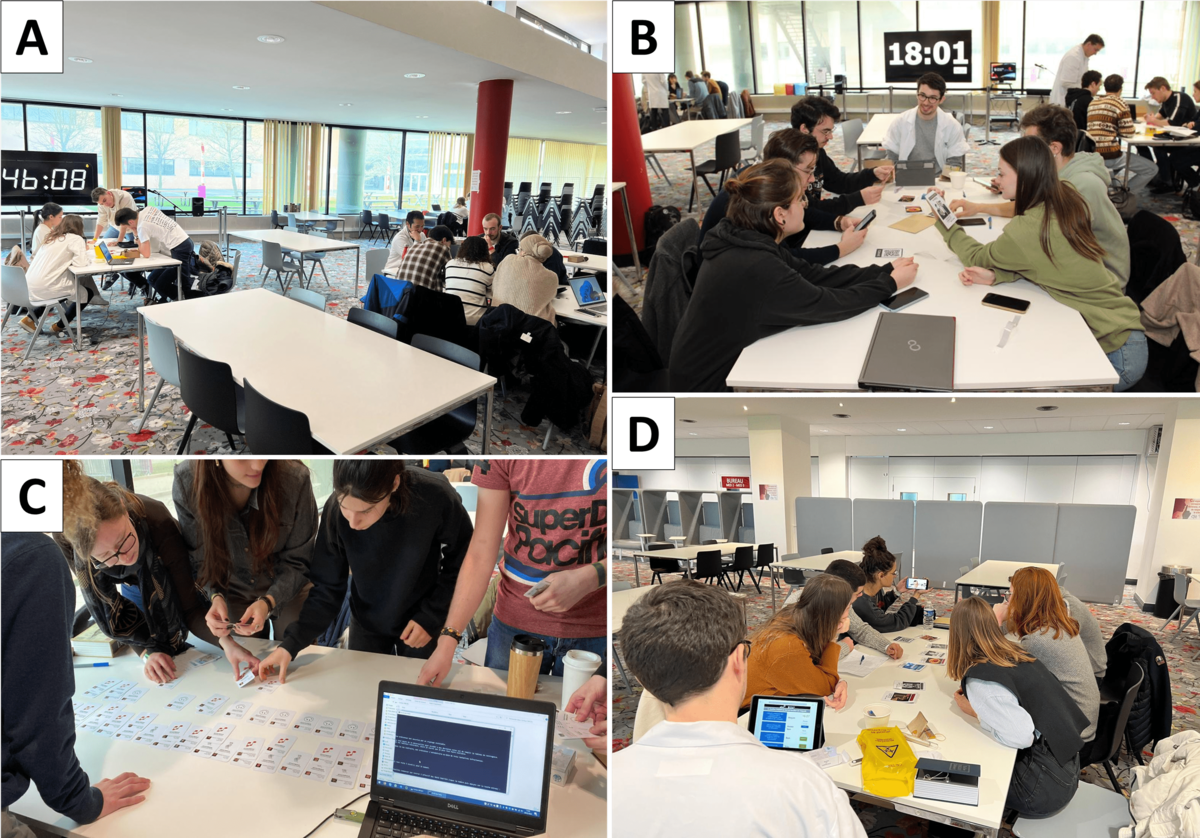
Scenes from the educational escape room illustrating the gameplay environment and student engagement. (A) Students collaborate in real time to solve puzzles under time pressure, with a visible countdown clock reinforcing immersion. (B) Small groups work simultaneously in the same session space, each supported by a facilitator in a white coat, simulating a clinical research environment. (C) Participants match test result cards to reconstruct the correct index and reference test pairs before entering the data into a contingency table via a terminal. (D) A team discusses the interpretation of diagnostic performance metrics from QR-coded test cards guided by the game’s unfolding narrative under the supervision of a dedicated faculty facilitator who performs real-time formative assessment using a tablet-based checklist. All individuals appearing in the photos provided written consent for their images to be used and published for educational and research dissemination purposes.

Twelve 120-minute sessions were conducted over the course of the program. Each session included a 15-minute introductory briefing, 60 minutes of gameplay, and 45 minutes of structured debriefing. Sessions were held in a large open space of approximately 150 m^2^, accommodating up to 45 students per session (Figure 3). Upon arrival, students were randomly assigned to teams of 4 to 6 using an algorithm triggered by swiping their university ID card.

Eight teams participated simultaneously in each session seated at individual tables spaced apart to allow for parallel problem-solving without interference. Although certain immersive elements were shared—such as a bookshelf, storage lockers with digital codes, a whiteboard, and a wall-mounted countdown timer—each team engaged independently with dedicated materials and instructions. All essential components were either prepacked in portable game bags (Figure 2) or duplicated in 8 identical sets to ensure autonomous progression. The briefing began with a short immersive video introducing the fictional mission and setting the narrative tone, aiming to engage students from the outset and foster a collaborative atmosphere (Multimedia Appendix 1).

Each session involved 9 facilitators: 1 game master assigned to each team and a session coordinator overseeing the entire room. Facilitators ensured smooth progression and conducted real-time formative assessments. After each puzzle, they rated the team’s clinical reasoning and collaborative dynamics using a tablet-based checklist with Likert scale items. This structure allowed students to work independently while maintaining consistent pedagogical oversight and documentation of performance. In total, 20 facilitators contributed across the sessions, representing a broad range of specialties including public health, radiology, nephrology, biology, biostatistics, pharmacology, pharmacy, pathology, family medicine, pediatrics, anesthesiology, and intensive care. All facilitators completed the escape room before student sessions to ensure fluency with the scenario and educational objectives.

To support scalability, all materials were designed for reuse. Game bags were reorganized after each session to allow for immediate reset. One month before implementation, the scenario was pilot-tested by 30 fourth-year medical students and 6 instructors whose feedback informed refinements to both content and logistics.

### Study Setting and Design

This monocentric prospective study was conducted between March 2023 and April 2023 at the Faculty of Medicine at the University of Lille All third-year medical students enrolled in the 2022‐2023 cohort were eligible to participate. The escape room was integrated into the mandatory curriculum as part of a course on research and critical appraisal and was a graded educational activity. Participation was required, and any student who missed a scheduled session was expected to provide a formal justification.

Before the session, participants provided demographic information (age range and sex) and reported previous experience with recreational escape games (“How many times have you participated in a recreational Escape Game?”). They also rated their comfort with critical appraisal of scientific articles using a 5-point Likert scale (“What is your level of comfort with critical appraisal of scientific articles?”) and submitted a free-text response identifying 3 words that best reflected their perception of critical appraisal (“Tell us the first three words that come to mind to describe your perception about critical appraisal of scientific articles”).

They then individually completed a knowledge questionnaire covering the 5 predefined learning objectives of the session. All questions were answered using “true” or “false” and grouped by objective. The same 5 learning objectives were reassessed during the debriefing immediately after the intervention using a different but equivalent set of “true”-or-“false” questions. A score out of 100 was calculated for each objective, and relative gain was defined as the absolute change between pre- and posttest scores divided by the pretest score. The complete list of questions is available in Multimedia Appendix 2. During the debriefing, students also completed an individual postintervention survey. They were asked to provide 3 words summarizing their perception of the escape room experience and to rate both the entertainment value and the pedagogical interest of the session on a scale from 0 to 10. They were again asked to rate their comfort with critical appraisal using the same 5-point Likert scale. In addition, they were asked the following: “Do you think an escape room is a suitable format for an introductory course on the critical appraisal of scientific articles?” Responses were collected using a 5-point Likert scale ranging from “Not at all suitable” to “Absolutely suitable.” Although “suitable” was not formally defined, the proximity of this question to those assessing the activity’s educational and entertainment value likely guided students to interpret it in terms of relevance, clarity, and pedagogical appropriateness. Finally, students were invited to leave open-ended comments about the session, including organizational aspects, perceived strengths, limitations, and suggestions for improvement.

This monocentric prospective study was conducted between March 2023 and April 2023 at the Faculty of Medicine at the University of Lille All third-year medical students enrolled in the 2022‐2023 cohort were eligible to participate. The escape room was integrated into the mandatory curriculum as part of a course on research and critical appraisal and was a graded educational activity. Participation was required, and any student who missed a scheduled session was expected to provide a formal justification.

### Ethical Considerations

The study protocol was reviewed and approved by the Institutional Review Board of the University of Lille in February 2023. The IRB determined that formal ethical approval was not required due to the pedagogical nature of the intervention and the exclusive use of anonymized data.

All procedures were conducted in accordance with the ethical standards of the institutional and national research committees and with the 1964 Helsinki Declaration and its later amendments or comparable ethical standards.

Upon registration on the study platform, students completed a short presession questionnaire and provided individual informed consent, including authorization to analyze their anonymized responses for research purposes. The study also ensured the privacy and confidentiality of all participants. Data were collected and stored on a secure, password-protected server, and identifying details, such as names and email addresses, were immediately deidentified and replaced with unique participant IDs to protect anonymity. No personal health information was collected.

Participants were not compensated for their involvement in the study (Multimedia Appendix 2).

### Statistical Analysis

Descriptive statistics were used to summarize participant characteristics and questionnaire responses. Quantitative variables were reported as means and SDs or medians and IQRs depending on their distribution. Categorical variables were expressed as counts and percentages. To assess knowledge gains, pre- and postintervention scores were compared using paired *t* tests. Associations between participant characteristics (age, sex, previous experience with escape rooms, and self-reported ease with critical appraisal) and relative score improvement were explored using univariate linear regression models. All statistical tests were 2-sided, and a *P* value of <.05 was considered statistically significant. All analyses were conducted using the R software (version 4.3.1; R Foundation for Statistical Computing).

### Qualitative Analysis of Free-Text Feedback

Students’ postintervention free-text comments were analyzed using the hierarchical classification method by Reinert [21], a lexical clustering approach based on word co-occurrence patterns. The analysis was conducted using the R Interface for Multidimensional Analysis of Texts and Questionnaires [22]. Comments were anonymized and preprocessed to remove low-frequency and noninformative words. The text corpus was segmented, and descending hierarchical classification was conducted to identify stable clusters of vocabulary. Factorial correspondence analysis was used to visualize the associations between lexical classes. The number of clusters retained was selected based on thematic coherence and interpretability. Two researchers (BLG and AH) independently reviewed and labeled each cluster using representative keywords and excerpts. Discrepancies were resolved through consensus. For reporting, all illustrative quotes were translated from French into English by the authors.

## Results

### Study Population

Of the 560 eligible third-year medical students, 530 (94.6%) participated in the escape room activity. Students who did not attend the escape room sessions were absent for justified personal or medical reasons. Of the 530 students who participated, 490 (92.5%) completed the preintervention questionnaire. Among them, 66.3% (325/490) were women, and 83.5% (409/490) were aged between 20 and 22 years. Most students reported limited or no previous experience with recreational escape rooms: 42% (206/490) had never participated in one, and 45.9% (225/490) had only done so once or twice. A substantial proportion expressed discomfort with critical appraisal of scientific literature: 18.8% (92/490) reported being “absolutely not comfortable,” and 33% (161/490) reported being “rather not comfortable” with the task (Table 1).

**Table 1.** Description of the study population (N=490).

Characteristic	Participants, n (%)
Female sex	325 (66.3)
Age group (y)	
<20	22 (4.5)
20‐22	409 (83.5)
22‐25	41 (8.4)
>25	18 (3.7)
Previous experience with recreational escape rooms	
None	206 (42)
1‐2 times	225 (45.9)
3‐4 times	52 (10.6)
≥5 times	7 (1.4)
Reported ease with critical appraisal of scientific articles	
Absolutely not comfortable	92 (18.8)
Rather not comfortable	161 (32.9)
Neither comfortable nor uncomfortable	201 (41)
Rather comfortable	31 (6.3)
Absolutely comfortable	5 (1)

### Escape Room Progression

**Figure 4.**
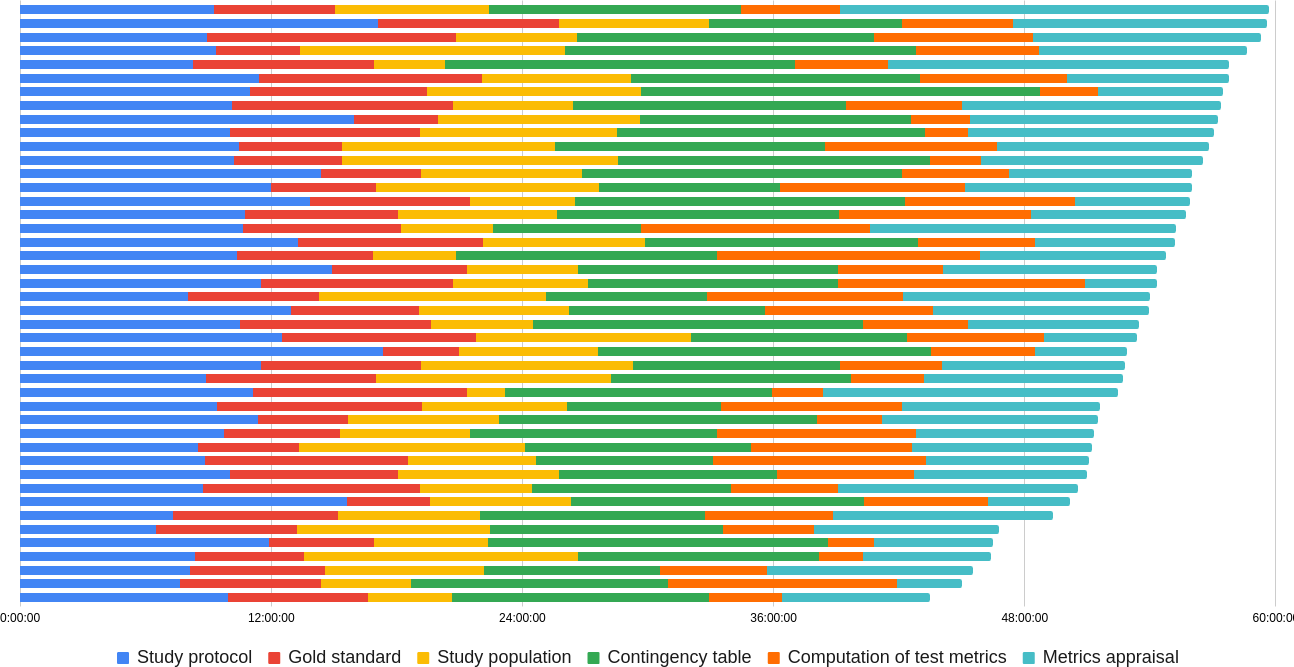
Puzzle progression timeline for a sample of 43 teams. Each horizontal line represents 1 team, and colors indicate the specific puzzle being solved at each moment during the session.

Divided into 96 teams across 12 sessions, students advanced through the escape room with a high degree of consistency. All teams completed the mission within the allotted time, with a mean duration of 53 (SD 4) minutes. Each of the 6 puzzles required approximately 10 minutes to solve. The fastest puzzle, focused on test metrics, was completed in an average of 6 minutes and 12 seconds (SD 2 min, 49 s), whereas the most time-consuming one, involving the construction of a contingency table, took 12 minutes and 21 seconds on average (SD 2 min, 51 s; (Table 2). Team progression was largely synchronous, with most groups working on the same puzzle simultaneously (Figure 4).

**Table 2.** Table 2. Completion time of puzzles.

Puzzle	Mean completion time (Minutes:seconds)	SD of mean completion time (Minutes:seconds)
Study protocol	10:52	2:35
Gold standard	7:11	2:09
Study population	7:31	2:41
Contingency table	12:21	2:49
Computation of test metrics	6:10	2:48
Metrics appraisal	9:15	3:24

### Pre- and Posttest Evaluations

**Figure 5.**
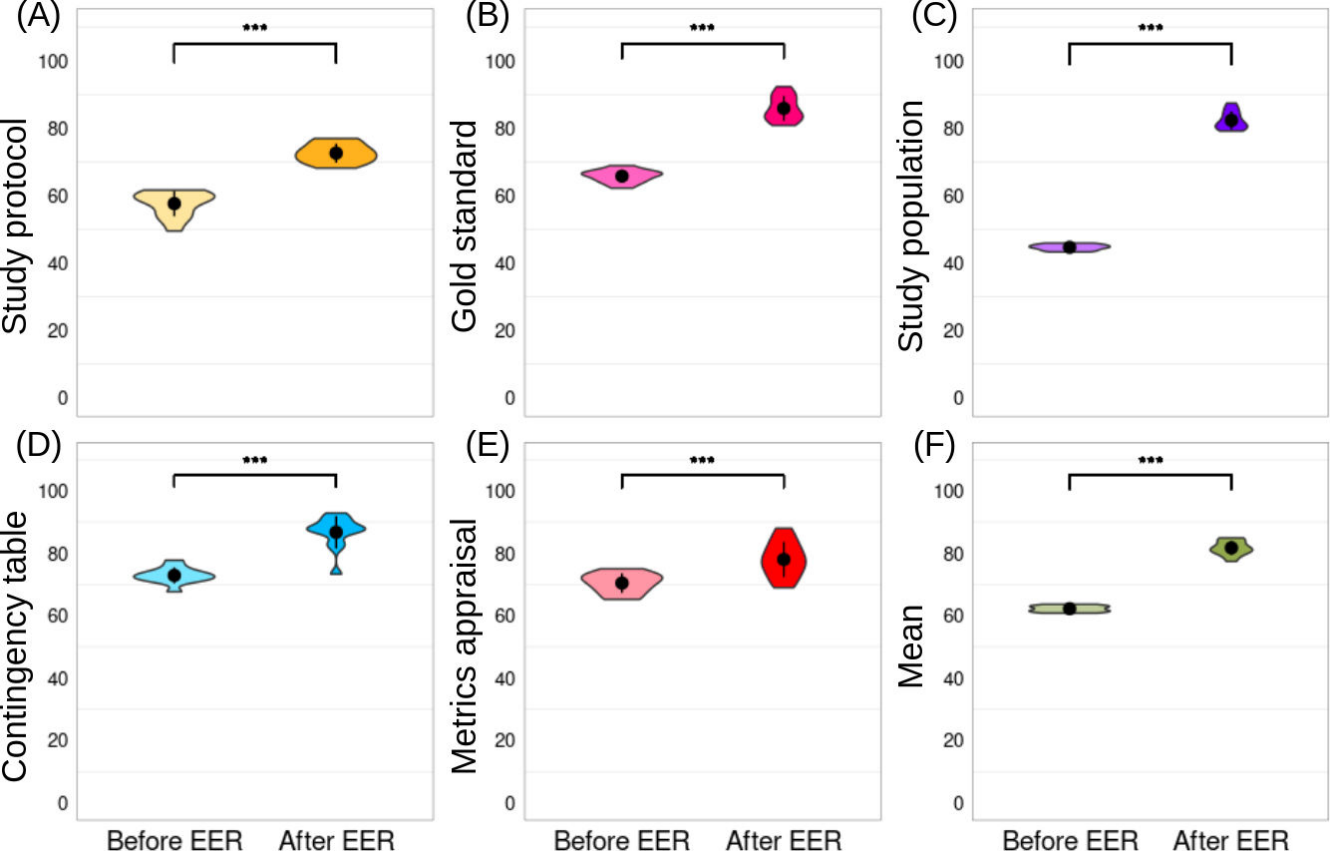
Pre- and posttest scores across learning objectives. Violin plots showing the distribution of student scores before and after the intervention for each of the 5 predefined learning objectives (top panels) and the overall mean score (bottom right panel). Asterisks indicate statistical significance. ***P<.001; EER: educational escape room.

A total of 85.3% (452/530) of the students completed the postintervention questionnaire. Student performance improved markedly following the intervention. The average score increased from 62/100 (SD 1) before the session to 82/100 (SD 2) afterward, representing a 32% gain (SD 5%; *P*<.001). Average subscores improved across all 5 learning objectives: from 58 (SD 3) to 71 (SD 3) for *study protocol*, from 74 (SD 2) to 89 (SD 4) for *gold standard*, from 43 (SD 1) to 83 (SD 3) for *study population*, from 71 (SD 2) to 88 (SD 5) for *contingency table*, and from 72 (SD 2) to 79 (SD 5) for *metric appraisal* (*P*<.001 in all cases; Figure 5). No significant associations were observed between score improvement and student characteristics (sex, age, previous escape room experience, or initial self-reported ease with critical appraisal), as shown in Table 3.

**Table 3.** Association between student characteristics and relative score improvement (univariate linear regressions).

Characteristic	β (95% CI)	*P* value
Sex (female vs male)	−2.5 (−6.8 to 1.9)	.26
Age category (ordinal scale)	1.7 (−2.1 to 5.4)	.38
Previous experience with ERs^a^ (ordinal scale)	0.9 (−2.8 to 4.6)	.63
Reported ease with critical appraisal of scientific articles (ordinal scale)	−0.6 (−3.3 to 2.1)	.69

a ER: escape room.

### Students’ Feedback

**Figure 6.**
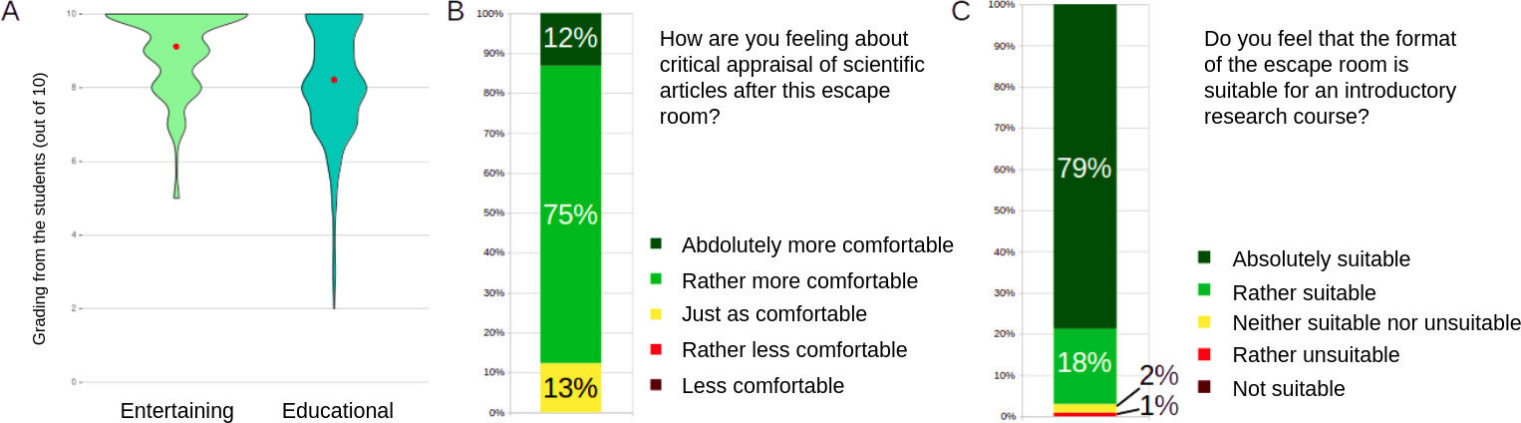
Students’ perceptions of the educational escape room (EER). (A) Student ratings of the EER’s entertainment and educational value (scale from 0‐10); red dots indicate mean scores. (B) Proportion of students reporting increased comfort with critical appraisal of scientific articles. (C) Students’ perception of the suitability of the escape room format as an introductory approach to research training.

Feedback was strongly favorable. Students rated the session highly in terms of enjoyment (mean 9.1/10, SD 1.1) and educational value (mean 8.2/10, SD 1.5). Most participants (393/452, 86.9%) reported feeling more comfortable with the appraisal of diagnostic accuracy studies after the session. Regarding the overall suitability of escape rooms for introducing research training, 79% (357/452) rated the format as “absolutely suitable” on a 5-point Likert scale (Figure 6). The term “suitable” was not explicitly defined but followed questions about entertainment and educational value, likely guiding interpretation in terms of relevance and clarity.

Students’ 3-word perceptions of research training shifted notably. Before the session, most descriptors were negative or reflected uncertainty (eg, “difficult,” “tedious,” “unknown,” or “complicated”). Afterward, the most frequent terms were markedly more positive—“engaging,” “entertaining,” “original,” and “educational” (Multimedia Appendix 3).

**Figure 7.**
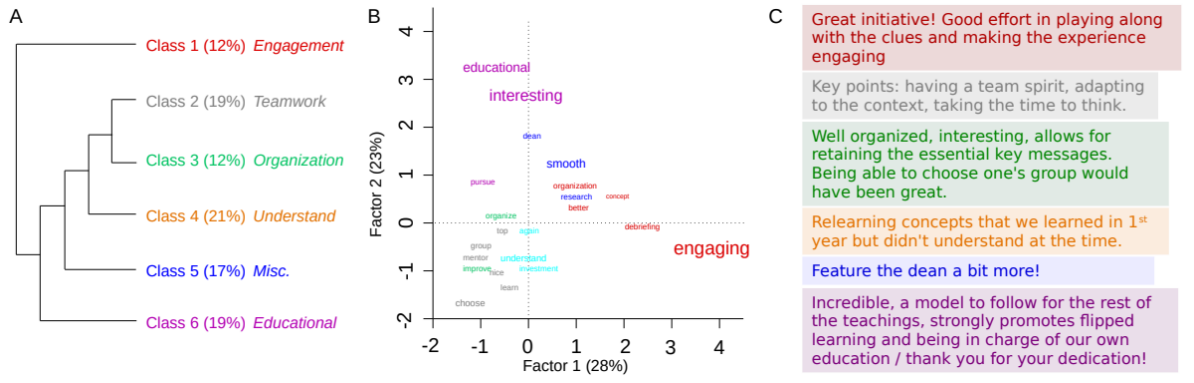
Classification and thematic mapping of students’ written feedback on the educational escape room. (A) The 6 semantic classes generated from a Reinert hierarchical clustering of students’ open-ended comments (translated from French by the authors). Each class reflects a distinct theme: engagement, teamwork, organization, understanding, educational value, and miscellaneous remarks. (B) The factorial correspondence analysis map of the vocabulary used across classes, with words spatially projected according to their association with the underlying dimensions (factor 1: 28%; factor 2: 23%). (C) Representative anonymized student quotes from each class highlighting the diversity of perspectives on the format, group dynamics, and perceived learning outcomes.

Thematic analysis of students’ free-text comments using the hierarchical classification by Reinert [21] identified 6 primary thematic clusters, reflecting engagement, teamwork, pedagogical value, conceptual understanding, and overall organization (Figure 7 [21]).

## Discussion

Our study demonstrates the feasibility of implementing a large-scale EER focused on introducing undergraduate medical students to core principles of diagnostic test evaluation as an entry point into research training. Student feedback echoed the 3 central aims of the intervention: to familiarize participants with key research concepts, foster teamwork, and improve perceptions of research-oriented courses. Pre- and postintervention assessments revealed significant immediate gains across all 5 targeted learning objectives. The activity was met with strong enthusiasm, in sharp contrast to students’ initial reservations about research training.

EERs have been scarcely used in recent years for medical students, mostly to teach various clinical disciplines such as emergency medicine [23,24], radiology [25,26], surgery [16], dermatology [18], pulmonology [27], pediatrics [28], internal medicine [29], general medicine [30], infectious diseases [31], physiology [32], and patient safety [33-35] (Table 4). In this paper, we report an introductory course designed for a transversal, nonclinical skill and with no expected prerequisite among undergraduate medical students. Another study designed an EER as an introductory course involving research articles with great success, although the aim of that study was to introduce a small group of participants to technical skills in surgery [16]. A recent study by Mirshahi et al [17] described a self-developed escape room designed to assess and reinforce research competencies across a multidisciplinary group of undergraduate and postgraduate health profession students, including medical students; while promising, this initiative involved a limited number of participants and did not focus specifically on diagnostic reasoning. Most studies published to date have involved relatively small cohorts of students (Table 4) (up to 245 students over 3 years). However, to consider real-life implementation of EERs within medical curricula, it is crucial to demonstrate their compatibility with the current growing size of medical classes, often comprising hundreds of students [36,37]. By using a parallelizable and repeatable gaming protocol, we showed the feasibility of delivering an innovative course to over 500 students while maintaining an entertaining and engaging environment. Another crucial challenge in implementing innovative pedagogic tools is the need to keep financial and human costs reasonable [38]. In our case, the allocated financial resources were constrained relative to the student count—€3.40 (US $3.95) per student. Through reusable material, this intervention can be replicated annually, refined, and adapted and enables the smoothing of the initial limited investment over the years [33]. As of July 2025, the EER has been repeated for 3 consecutive years (approximately 1800 students) without any additional financial cost. On the other hand, the human resources required for our intervention were substantial, with a total of 20 supervisors involved across the 12 sessions.

**Table 4.** Literature review of educational escape room experiments for medical students.

Study	Country	Title	Sample size	Specialty	Student level	Objective	Type of escape room	Evaluation
‍Zhang et al [23], 2018	United States	“Trapped as a Group, Escape as a Team: Applying Gamification to Incorporate Team-building Skills Through an ‘Escape Room’ Experience”	10	Emergency medicine	Residents	Team-building exercise	Commercial escape room	Postevent satisfaction survey
Backhouse and Malik [33], 2019	United Kingdom	“Escape Into Patient Safety: Bringing Human Factors to Life for Medical Students”	19	Specialty choice module	Undergraduate	Patient safety teaching	Self-created suitcase-based escape room	After-action review
Diemer et al [34], 2019	United States	“Patient Safety Escape Room: A Graduate Medical Education Simulation for Event Reporting”	120	Patient safety	Residents	Patient safety hazard reporting	Hospital case–based escape room	Postevent satisfaction survey
Kinio et al [16], 2019	Canada	“Break out of the Classroom: The Use of Escape Rooms as an Alternative Teaching Strategy in Surgical Education”	13	Surgery	Undergraduate	Motivation, engagement, and satisfaction	Escape room stations in the simulation center	Postevent satisfaction survey
‍Zhang et al [35], 2019	United States	“Finding the 'QR’ to Patient Safety: Applying Gamification to Incorporate Patient Safety Priorities Through a Simulated ‘Escape Room’ Experience”	130	General medicine	Residents	Patient safety teaching	Escape room stations in the simulation center	Postevent survey
Jambhekar et al [25], 2020	United States	“Benefits of an Escape Room as a Novel Educational Activity for Radiology Residents”	164	Radiology	Residents	Team-building exercise	Self-created portable escape room	Postevent satisfaction survey
Guckian et al [18], 2020	United Kingdom	“Exploring the Perspectives of Dermatology Undergraduates With an Escape Room Game”	16	Dermatology	Undergraduate	Improving students’ perceptions on the field	Self-created immersive escape room	Postevent satisfaction survey and focus groups
Liu et al [26], 2020	United Kingdom	“Feasibility of a Paediatric Radiology Escape Room for Undergraduate Education”	19	Pediatric radiology	Undergraduate	Knowledge and satisfaction	Self-created portable escape room	Pre- and posttest and satisfaction surveys
Abensur Vuillaume et al [24], 2021	France	“A Didactic Escape Game for Emergency Medicine Aimed at Learning to Work as a Team and Making Diagnoses: Methodology for Game Development”	10	Emergency medicine	Health care workers	Team-building exercise	Self-created immersive escape room	Not specified
Khanna et al [29], 2021	United States	“Escape MD: Using an Escape Room as a Gamified Educational and Skill-Building Teaching Tool for Internal Medicine Residents”	86	Internal medicine	Residents	Team work, critical thinking, and communication skills	Self-created immersive escape room	Postevent satisfaction survey
Akatsu et al [30], 2022	Japan	“Teaching ‘medical interview and physical examination’ from the very beginning of medical school and using ‘escape rooms’ during the final assessment”	140	General medicine (interview and physical examination)	Undergraduate	Course final assessment	Game-based scenarios with simulators	Postevent satisfaction survey
Dimeo et al [31], 2022	United States	“A Virtual Escape Room versus Lecture on Infectious Disease Content: Effect on Resident Knowledge and Motivation”	30	Infectious disease	Residents	Knowledge and motivation	Virtual escape room	Pretest, posttest, and motivation evaluations
Carrasco-Gomez et al [32], 2023	Spain	“Impact of a Peer-to-Peer Escape Room Activity in the Learning of Human Physiology of Medical Students From the University of Málaga”	245	Physiology	Undergraduate	Human physiology knowledge	Peer-to-peer–designed escape room	Comparative scores (vs nonparticipants) and postevent satisfaction survey
Fedorcsak [20], 2024	Norway	“Moderate Benefit of Escape Room Game on Learning Outcome in Medicine”	213	REI^a^	Undergraduate	General knowledge in REI	Self-created immersive escape room	Postevent comparative scores (vs nonparticipants)
Mirshahi et al [17], 2025	Iran	“‘MORAD ESCAPE,’ a Novel Research-Based Escape Room Approach for Evaluating Research Competencies of Health Professions Students”	60	Research competencies	Undergraduate and postgraduate	Research competency checklist	Self-created immersive escape room (escapED program)	Postevent evaluation of research competency and satisfaction survey

a REI: reproductive endocrinology and infertility.

Whether EERs meaningfully improve learning outcomes remains uncertain [12,15,19,20]. Previous studies have reported mixed results, with some suggesting potential benefits [32] and others, such as a recent controlled study by Fedorcsak [20], finding only modest improvements in declarative knowledge after a single EER session (Cohen *d*=0.22). In our study, we observed an immediate gain across 5 targeted learning objectives. However, in the absence of a control group and long-term follow-up, these findings should be interpreted cautiously. The structured, sequential format of the EER, aligned with the logic of a diagnostic study, may have supported knowledge acquisition, but the true impact on learning remains difficult to disentangle from engagement effects or short-term memory recall. Importantly, the intervention appeared accessible across subgroups, with no influence of previous escape room experience or self-perceived ease with critical appraisal. Beyond performance, the ability to foster interest in research methodology among a large cohort of students may in itself constitute a valuable educational outcome [12,15,19].

Our findings suggest a strong alignment between student feedback and our initial pedagogical intentions: namely, to introduce foundational research skills, foster collaborative teamwork, and demystify research training through an engaging and enjoyable format. Motivating undergraduate medical students to engage with research remains a recognized challenge [7,8,10]. In our study, students reported a marked shift in perception, from predominantly negative views on research training to positive reflections on the escape room experience. Free-text responses frequently emphasized the value of teamwork, a dimension often underrepresented in their curriculum, as well as the quality of facilitation and meaningful interactions with faculty. As observed in other EER-based interventions, we noted a high level of student cooperation and an intrinsic drive to complete the challenge within the allotted time [16,18,25,29,31]. This positive, collaborative atmosphere appeared to promote rich student-mentor interactions, an important benefit for a generation of learners shaped by recent experiences of social distancing and remote education [39].

This study has several limitations. As a pilot implementation, it was not designed to isolate the specific effects of the escape room format compared to traditional pedagogical approaches. The absence of a control group precludes any definitive conclusions about the causal impact of the intervention on student performance or attitudes. All outcome measures relied on self-reported or short-term evaluations, with no assessment of knowledge retention over time. Moreover, the educational content focused exclusively on diagnostic accuracy studies. While this choice was pedagogically motivated, it limits the generalizability of the findings to other types of research designs. This study was also conducted in a single institution, which may affect broader applicability. Future research should explore the added value of EERs in research training using comparative designs—such as cluster randomized trials—and include a broader range of research topics and longer-term follow-up to assess sustained learning outcomes. Despite these limitations, this study benefits from a high participation rate, detailed process documentation, and rich qualitative feedback, which collectively provide meaningful insights into the feasibility, perceived value, and immediate educational impact of this innovative teaching format.

In conclusion, this study demonstrates the feasibility of a large-scale, low-cost, and replicable EER to introduce undergraduate medical students to key concepts of diagnostic test evaluation. The format was well received and may offer a promising entry point into research training provided that its targeted scope and pedagogical objectives are clearly defined.

## Supplementary material

10.2196/71339 Multimedia Appendix 1 Immersive mission briefing shown before the start of the escape room. This introductory video was designed to immerse students in a fictional investigation scenario. It presented the mission’s narrative context, introduced the main characters and storyline, and created a suspenseful atmosphere to enhance student engagement from the outset. The video served solely to set the scene and did not provide any instructions or clues related to the upcoming puzzles.

10.2196/71339 Multimedia Appendix 2 Content of the “true”-or-“false” questionnaire administered immediately before and after the escape room intervention. Items are grouped by learning objective (*study protocol*, *study population*, *gold standard*, *contingency table*, and *metric appraisal*). For each pretest item, the expected correct answer and the proportion of students who responded correctly at baseline are reported; identical or conceptually equivalent items were presented at the posttest time point to assess immediate knowledge gain.

10.2196/71339 Multimedia Appendix 3 Word clouds illustrating students’ perceptions before and after the educational escape room (EER). The left panel reflects students’ initial impressions of critical appraisal of scientific articles before the intervention, whereas the right panel captures their reactions to the EER format afterward. Comments were originally written in French and translated by the authors.

## Abbreviations

EER: educational escape room
