# Supplementary material for: Engaging Undergraduate Medical Students With Introductory Research Training via an Educational Escape Room: Mixed Methods Evaluation
Source: JMIR Med Educ. 2025 Dec 8;11:e71339. doi: 10.2196/71339 (PMC12685230; doi:10.2196/71339)
Supplement: Multimedia Appendix 2 [file mededu-v11-e71339-s002.docx]

**Supplementary material**

**Supplementary Table 1. Content of the true/false questionnaire administered immediately before and after the escape‑room intervention.**

*Items are grouped by learning objective (Study Protocol, Study Population, Gold Standard, Contingency Table, Metrics Appraisal). For each pre‑test item, the expected correct answer and the proportion of students who responded correctly at baseline are reported; identical or conceptually equivalent items were presented post‑test to assess immediate knowledge gain.*

| **Objective** | **Pre-test** | **Expected answer** | **Accuracy (%)** |
| --- | --- | --- | --- |
| Study protocol | A diagnostic study aims at identifying risk factors for a pathology. | False | 78.8 |
|  | A diagnostic study aims at identifying a diagnostic test superior to the reference test. | False | 45.5 |
|  | A diagnostic study aims at measuring the discriminative abilities of a diagnostic test under study. | True | 51.8 |
| Study Population  ‍ | A diagnostic study requires having comparable groups at the beginning of the study. | False | 22.2 |
|  | Confounding factors are common due to the observational nature of diagnostic studies. | False | 66.1 |
| Gold standard | The reference test is also called the gold standard. | True | 89.6 |
|  | The gold standard must be clearly described by the authors. | True | 62.4 |
|  | The gold standard allows for the classification of patient groups at the beginning of the diagnostic study. | True | 28.6 |
|  | Only its reliability matters in the choice of the gold standard. | False | 93.9 |
|  | The gold standard is never responsible for classification bias. | False | 97.3 |
| Contingency table | Sensitivity is an intrinsic characteristic of a test. | True | 87.3 |
|  | Positive likelihood ratio is an intrinsic characteristic of a test. | True | 6.1 |
|  | Negative predictive value is an intrinsic characteristic of a test. | False | 82.7 |
|  | Specificity is an intrinsic characteristic of a test. | True | 81.2 |
|  | Prevalence is an intrinsic characteristic of a test. | False | 88.8 |
|  | The specificity of the test can affect its sensitivity. | False | 77.6 |
|  | The test's positivity threshold can affect its sensitivity. | True | 46.9 |
|  | The prevalence of the disease being diagnosed can affect the sensitivity of the test under study. | False | 41.6 |
|  | Population characteristics of the study can affect the sensitivity of the test under study. | True | 38.2 |
|  | The negative predictive value of the test can affect its sensitivity. | False | 80.6 |
|  | A highly sensitive test has few false negatives. | True | 38.8 |
|  | Sensitivity varies depending on the specificity of the test under study. | False | 83.5 |
|  | The higher the sensitivity, the better the test correctly detects cases. | True | 66.3 |
|  | Sensitivity increases with the prevalence of the disease. | False | 74.1 |
|  | Sensitivity is an intrinsic property of the test. | True | 71.0 |
| Metrics Appraisal | In case of a positive urea breath test, your patient must necessarily undergo a gastric endoscopy. Given the invasive nature of this examination, sensitivity should be favored for the urea test. | False | 31.2 |
|  | In case of a positive urea breath test, your patient must necessarily undergo a gastric endoscopy. Given the invasive nature of this examination, negative predictive value should be favored for the urea test. | False | 80.0 |
|  | In case of a positive urea breath test, your patient must necessarily undergo a gastric endoscopy. Given the invasive nature of this examination, specificity should be favored for the urea test. | True | 30.6 |
|  | In case of a positive urea breath test, your patient must necessarily undergo a gastric endoscopy. Given the invasive nature of this examination, the negative likelihood ratio should be favored for the urea test. | False | 84.4 |
|  | In case of a positive urea breath test, your patient must necessarily undergo a gastric endoscopy. Given the invasive nature of this examination, the p-value should be favored for the urea test. | False | 97.4 |
|  | **Post-test** |  |  |
| Study protocol | Evaluating the superiority of the test under study over the gold standard is an objective of a diagnostic study. | False | 87.2 |
|  | Predicting a patient's treatment response is an objective of a diagnostic study. | False | 97.0 |
|  | Estimating the risk of mortality in a population of patients is an objective of a diagnostic study. | False | 94.0 |
|  | Evaluating a test's ability to distinguish between diseased and non-diseased subjects is an objective of a diagnostic study. | True | 84.2 |
|  | Evaluating the agreement of the test under study with the results of the reference test for a given disease is an objective of a diagnostic study. | True | 68.4 |
| Study Population | The study population for a diagnostic study must necessarily include both diseased and non-diseased subjects. | True | 97.4 |
|  | The groups of diseased and non-diseased subjects must be comparable. | False | 81.8 |
|  | The distinction between the two groups in the study population is ensured by the test under study. | False | 72.2 |
|  | The interpretation of results in a diagnostic study is often limited by confounding factors. | False | 92.3 |
|  | Selection bias is never found in diagnostic studies. | False | 61.7 |
| Gold Standard | The gold standard must necessarily result in a definitive diagnosis. | False | 87.4 |
|  | The gold standard must be reliable and reproducible. | True | 100 |
|  | The gold standard is only conducted within the population of diseased subjects. | False | 96.8 |
|  | A diagnostic study can proceed without using a gold standard. | False | 97.3 |
|  | The gold standard is essential for creating a contingency table. | True | 78.8 |
| Contingency Table | Patients with a positive test among the diseased are false positives. | False | 96.2 |
|  | Non-diseased patients with a positive test are false positives. | True | 91.9 |
|  | Diseased patients with a negative test are false positives. | False | 100 |
|  | Diseased patients among the positive tests are false positives. | False | 97.4 |
|  | Non-diseased patients among the positive tests are false positives. | True | 42.9 |
| Metrics Appraisal | A highly specific test (near 100%) with a positive result can exclude the possibility that the patient is diseased. | False | 70.9 |
|  | A highly specific test (near 100%) with a positive result is strongly suggestive of a positive diagnosis in the patient. | True | 57.1 |
|  | A highly specific test (near 100%) with a positive result can indicate that this test (or clinical sign) is almost pathognomonic. | True | 78.9 |
|  | A highly specific test (near 100%) with a positive result can conclude with almost no false negatives. | False | 67.0 |
|  | A highly specific test (near 100%) with a positive result can justify initiating a potentially burdensome treatment for the patient. | True | 81.1 |
|  | Literature data confirm that the most important prognostic factor in sepsis is the prompt administration of antibiotic. When considering a new diagnostic test biomarker, sensitivity should be prioritized. | True | 66.7 |
|  | Literature data confirm that the primary prognostic factor in sepsis is the speed of antibiotic administration. When considering a new diagnostic test biomarker, specificity should be prioritized. | False | 85.0 |
|  | Literature data confirm that the primary prognostic factor in sepsis is the speed of antibiotic administration. When considering a new diagnostic test biomarker, positive predictive value should be prioritized. | False | 88.9 |
|  | Literature data confirm that the primary prognostic factor in sepsis is the speed of antibiotic administration. When considering a new diagnostic test biomarker, the false positive rate should be prioritized. | False | 96.8 |
|  | Literature data confirm that the primary prognostic factor in sepsis is the speed of antibiotic administration. When considering a new diagnostic test biomarker, the disease prevalence should be prioritized. | False | 97.3 |
