# Supplementary figures and images for: Engaging Undergraduate Medical Students With Introductory Research Training via an Educational Escape Room: Mixed Methods Evaluation
Source: JMIR Med Educ. 2025 Dec 8;11:e71339. doi: 10.2196/71339 (PMC12685230; doi:10.2196/71339)

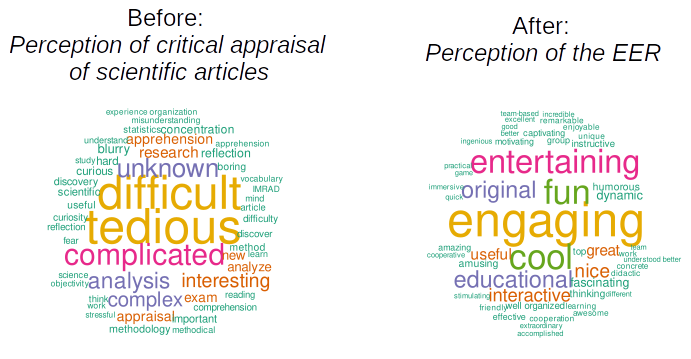

Supplement: Multimedia Appendix 3 [file mededu-v11-e71339-s003.png]
